# Hemolymph proteome changes during worker brood development match the biological divergences between western honey bees (*Apis mellifera*) and eastern honey bees (*Apis cerana*)

**DOI:** 10.1186/1471-2164-15-563

**Published:** 2014-07-05

**Authors:** Mao Feng, Haitham Ramadan, Bin Han, Yu Fang, Jianke Li

**Affiliations:** Institute of Apicultural Research/Key Laboratory of Pollinating Insect Biology, Ministry of Agriculture, Chinese Academy of Agricultural Science, Beijing, 100093 China

**Keywords:** *Apis mellifera ligustica*, *Apis cerana cerana*, Larvae, Pupae, Hemolymph, Proteome

## Abstract

**Background:**

Hemolymph plays key roles in honey bee molecule transport, immune defense, and in monitoring the physiological condition. There is a lack of knowledge regarding how the proteome achieves these biological missions for both the western and eastern honey bees (*Apis mellifera* and *Apis cerana*). A time-resolved proteome was compared using two-dimensional electrophoresis-based proteomics to reveal the mechanistic differences by analysis of hemolymph proteome changes between the worker bees of two bee species during the larval to pupal stages.

**Results:**

The brood body weight of *Apis mellifera* was significantly heavier than that of *Apis cerana* at each developmental stage. Significantly, different protein expression patterns and metabolic pathways were observed in 74 proteins (166 spots) that were differentially abundant between the two bee species. The function of hemolymph in energy storage, odor communication, and antioxidation is of equal importance for the western and eastern bees, indicated by the enhanced expression of different protein species. However, stronger expression of protein folding, cytoskeletal and developmental proteins, and more highly activated energy producing pathways in western bees suggests that the different bee species have developed unique strategies to match their specific physiology using hemolymph to deliver nutrients and in immune defense.

**Conclusions:**

Our disparate findings constitute a proof-of-concept of molecular details that the ecologically shaped different physiological conditions of different bee species match with the hemolymph proteome during the brood stage. This also provides a starting point for future research on the specific hemolymph proteins or pathways related to the differential phenotypes or physiology.

**Electronic supplementary material:**

The online version of this article (doi:10.1186/1471-2164-15-563) contains supplementary material, which is available to authorized users.

## Background

Like other arthropod insects, the honey bee has an open circulatory system containing hemolymph that surrounds its tissues [1]. Similar to the blood in vertebrates [2], the insect hemolymph plays a role in both immune defense and primary energy storage. Its defensive role is achieved by antimicrobial factors produced largely by the fat body and, to a lesser extent, hemocytes, which inhibit growth of microorganisms via an encapsulation pathway. As the system responsible for transporting various molecules throughout the body (such as nutrients, ions, and hormones), hemolymph also mirrors the physiological condition of the body, making it suitable for monitoring systemic changes in other pathways [3]. Hemolymph is transparent or light yellowish color and represents about 25-30% of the body weight of honey bee at the hatching stage, decreasing as their age increases [1, 4, 5]. In contrast to vertebrate blood, the honey bee hemolymph has relatively high concentrations of inorganic ions, amino acids, sugars, proteins, and many other substances, but its contents are varied in regards to developmental stages, sex, and season [6, 7].

Due to its roles in molecule transportation and immune defense, characterization of the hemolymph proteome is of vital importance in understanding its function within the honey bee. Several works concerning the molecular functions of honey bee hemolymph have been reported so far. For instance, the hemolymph proteome of larvae, pupae, and adults, as well as a differential comparison of the hemolymph proteome between the queen and worker, have been characterized [1, 8–10]. In addition, the biochemical components and biological activities of honey bee hemolymph have been documented [4, 10, 11]. All of these works are focused only on the western honey bee (*Apis mellifera*), and there have been no works examining the eastern honey bee (*Apis cerana*). Despite their similarities as cavity-nesting species, their native ranges do not overlap; western bees have a large and diverse native range, spanning Europe, Africa, and the Middle East, while the eastern bees are exclusive to the Asian continent [12]. During the colonization of their current ecological ranges, both species have experienced strong selection for adaptations [13]. Therefore, large differences in physiology and behavior exist between the western and eastern honey bees. *Apis cerana* workers have smaller body sizes and start working earlier in the day than *Apis mellifera* workers (due to larger body size of the latter) and they can survive extreme fluctuations in ambient temperature and long periods of rainfall [14]. *Apis cerana* is more industrious in collecting nectar from scattered flowers, while *Apis mellifera* worker has stronger foraging capacity of large flower patches [15]. Moreover, the eastern bees have evolved unique biological characteristics in resisting both wasps and the ectoparasitic mite, *Varroa destructor,* while most western bees are susceptible to both [16, 17]. Although most of the above discussed differences in biological parameters of these two bee species are known to have a genetic basis, all must ultimately a manifestation of changes in protein expression and/or pathway. The biological divergences between the brood stages of western and eastern bees still remain unknown.

*Apis cerana cerana* (Acc), native to China, is one of the major ecotypes of the eastern honey bee. With over three million colonies, Acc is important for the beekeeping industry in China and throughout Asia for honey production [18], as well as for crop pollination and maintaining biodiversity in the ecosystem. Despite its economic and ecological importance, investigation of the biological characteristics of Acc, particularly in molecular research, is lagging far behind what is known of its western counterpart. Only recently has research on Acc expanded beyond the description of behaviors or social traits to a molecular level; researchers have examined differential gene expression profiling between the queens and workers [19], the sequencing of the mitochondrial genome [20], creation of a linkage map of single nucleotide polymorphisms (SNP), and the pathological basis of Chinese sacbrood disease (CSD) in this species [12]. Until now, few works have reported on biological differences between the western and eastern worker bees, such as the comparison of molecular olfactory mechanisms [9, 21] and dance behavior [22].

The development of a honey bee larva to a pupa is a crucial life transition. In the first six days, the larvae increase in body weight by as much as 1500 times. This astounding speed of development demands a high supply of nutrients transported from the hemolymph. During the initial period of development, especially in the first 48 h, the brood immunity, is very fragile and easily susceptible to a pathological invasion such as *Paenibacillus larvae* [3]. Brood development occurs within the confines of a comb cell; the brood has limited ability to move away from any invading pathogen, nor can it use behavioral mechanisms as adult bees do [23]. To deal with this challenge and to ensure normal growth, the hemolymph of honey bee brood functions as cellular and humoral mechanisms of defense [2]. Such changes must involve a cascade of events that regulate the expression of the hemolymph proteome. Although our previous work has characterized the hemolymph proteome alteration during almost the complete brood developmental stage in western worker bees [9], knowledge of the hemolymph proteomic differences between western and eastern bees during the brood stage is still lacking. To fill this gap, hemolymph proteomic differences were compared across the larval to pupal developmental stages between the western and eastern bees. This will provide new insight into how the hemolymph can help the different bee species in achieving their normal ontogenetic development of brood.

## Results

### Brood body weight comparison of Aml and Acc workers

**Figure 1:**
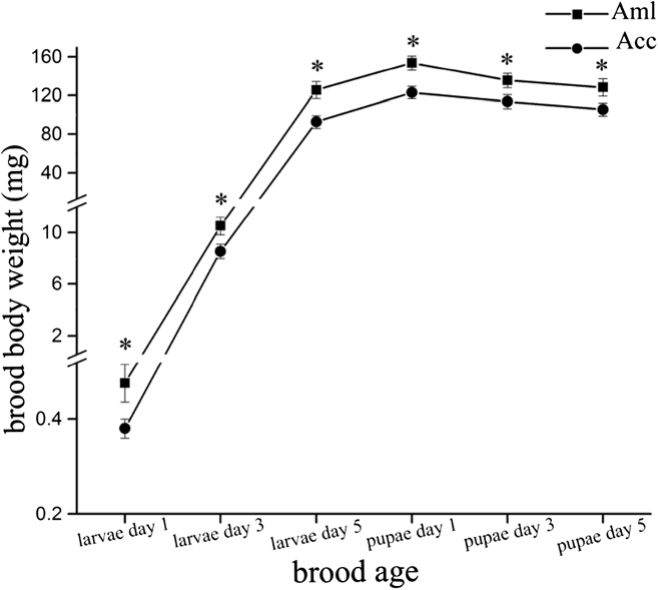
**Brood body weight at different developmental time points represents as mean ± SE (n =200).** The upper line is *A. m. ligustica* (Aml) and the lower line is *A. c. cerana* (Acc). Asterisks indicate statistically significant differences (*p* < 0.05).

The body weight of 200 larvae and pupae samples were measured at six time-points each from both bee species. It was shown that Aml brood possessed a significantly heavier body weight than that of Acc at each developmental stage (*p* < 0.05) (Figure 1).

### Qualitative comparisons of differentially expressed proteins

**Figure 2:**
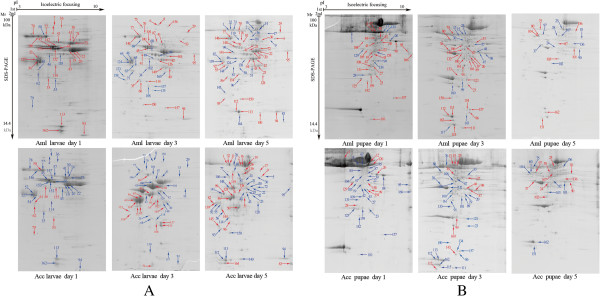
**2-DE profiles of hemolymph proteome. A** represents the gel images of honey bee worker (*A. m. ligustica* and *A. c. cerana*) larvae on days 1, 3 and 5, respectively. **B** is the gel images of honey bee worker pupae on days 1, 3 and 5, respectively. Differentially expressed protein spots of known identity are marked with color codes. The red labels indicate the proteins that were upregulated in Aml and downregulated in Acc, while the blue labels indicate upregulated in Acc and down-regulated in Aml on days 1, 3 and 5 old larvae and pupae, respectively.

**Figure 3:**
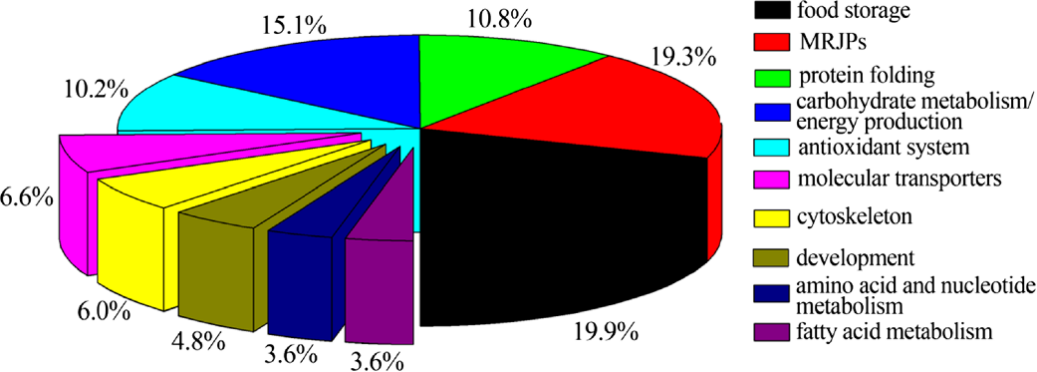
**Protein functional categorization of differentially expressed hemolymph proteins of both Aml and Acc honey bee workers at larval (days 1, 3, and 5) and pupal (days 1, 3, and 5) stages.** Pie chart of each protein groups on the basis of GO analysis, the percentage is obtained based on the number of successfully identified protein spots under each Go terms.

**Figure 4:**
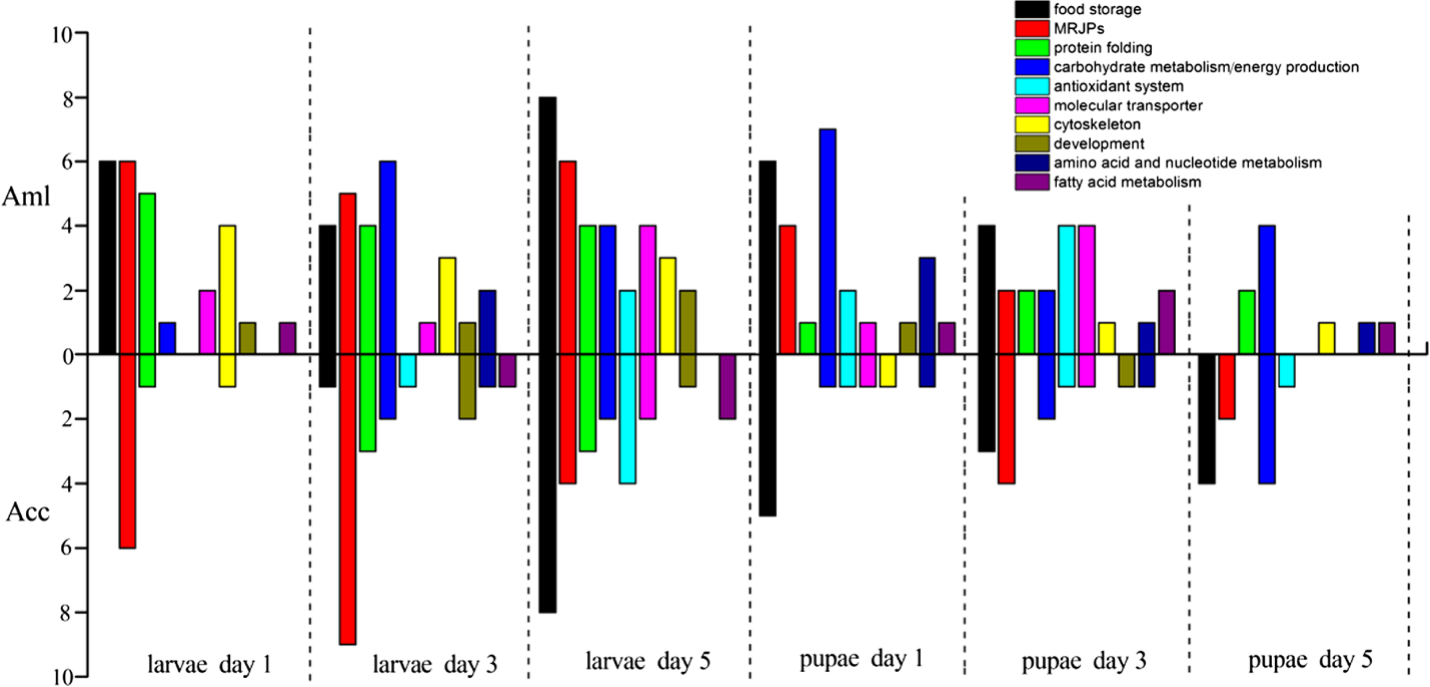
**Comparisons of the upregulated protein spots of each GO terms in the hemolymph of both** ***A. m. ligustica*** **(Aml) and** ***A. c. cerana*** **(Acc) honey bee workers at larval and pupal stages.**

To compare the difference between the hemolymph proteomes of Aml and Acc workers, 2-DE images of six time-points across larval to pupal stages were established (Figure 2). In order to keep consistency with our previous study [9], we chose the same six time-points of brood at a particular age for sampling and ran these new sample sets with similar amounts of hemolymph proteins. In general, there was good reproducibility of the resolved proteins on 2-DE gel images of Aml hemolymph between this study and our previous work. Over 440 protein spots were detected on each of these 2-DE images. The corresponding spots were compared between the two species at each of the respective developmental stages. We found the following number of protein spots demonstrated a significant change of abundance (fold change >1.5, *p* < 0.05) at each time point: larvae 1 day = 36, larvae day 3 = 58, larvae day 5 = 70, pupae day 1 = 48, pupae day 3 = 49 and pupae day 5 = 21. Of these differentially detected protein spots we identified 34, 47, 59, 36, 40, and 20 at day 1, day 3, and day 5 larvae and day 1, day 3 and day 5 pupae by LC-MS/MS, respectively (Additional file 1: Table S1; Figure 2). The remaining protein spots were not identified because their low abundance did not produce enough spectra, or because the database search scores yielded ambiguous results.The 166 differentially expressed protein spots were identified as 74 non-redundant proteins. These spots were annotated into 10 GO terms. Five protein groups were overrepresented in both bee species: food storage (19.9%), major royal jelly proteins (19.3%), protein folding (10.8%), carbohydrate metabolism/energy production (15.1%), and antioxidant activity (10.2%) (Figure 3). Although the proteins that were differentially expressed between the two bee species were annotated to the similar protein groups, Aml, in general, had a higher number of abundantly expressed proteins involved in carbohydrate metabolism/energy production, protein folding, molecular transporter, and amino acid metabolism at all six time points than the Acc (Figure 4). Only MJRPs and food storage were more abundantly expressed in Acc than in Aml, MRJPs were in larval stages of day 3 and pupal stages of day 3 and 5, while food storage was only in pupal stage of day 5 (Figure 4).

### Quantitative comparisons of differentially expressed proteins

**Figure 5:**
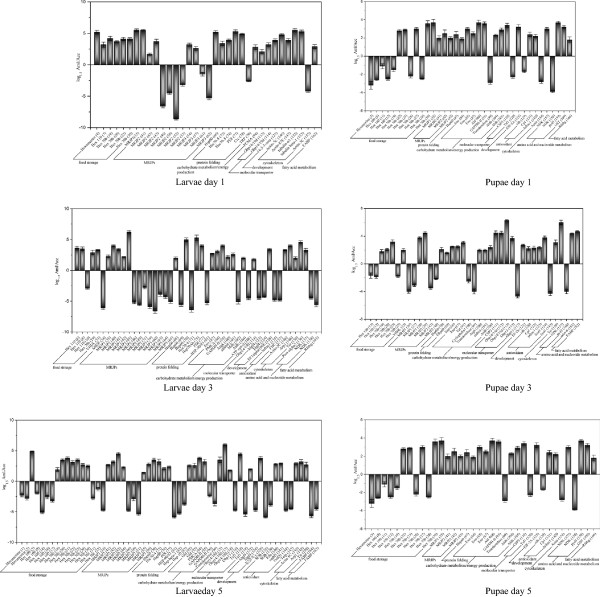
**Quantitative comparisons of the total differentially expressed hemolymph proteins of both** ***A. m. ligustica*** **(Aml) and** ***A. c. cerana*** **e (Acc) worker bee larvae and pupae at development time points on days 1, 3, and 5 respectively.** The ratios of the protein abundance (Aml/Acc) are transformed, and the protein spots with |log_1.5_ ratio| >1 (*p* < 0.05) are selected as the differentially expressed proteins. Protein names (in abbreviations) and protein numbers (in the parenthesis) are listed as in the Additional file 1: Table S1. Positive value indicates higher expression in Aml and negative values denote higher expression proteins in Acc.

To better understand the biological significance of each protein, the expressional intensity of each protein was generated in terms of its volume (Additional file 2: Table S2) and then compared between the two bee species using log ratio (log_1.5_ Ratio) *t*-test. The p-values for the differentially expressed proteins (*p* < 0.05) were calculated as the ratio of the protein abundance (Aml/Acc). As the two bees share similar complements of proteins, this approach of protein quantification was particularly effective for comparing protein expression levels between them. This comparison could help to estimate the extent of biological significance of each protein (Figure 5).

In day 1 larva, 9 proteins had stronger expressional levels (log_1.5_ Ratio ≥ 4.5) in Aml, and 4 were stronger in Acc (Additional file 1: Table S1; Figure 5). Even though a large number of protein spots were upregulated in day 3 larva of Aml, only 8 proteins had stronger expressional levels (log_1.5_ Ratio ≥ 4), while 18 were more strongly expressed in Acc (Additional file 1: Table S1; Figure 5). This was similar in the day 5 larvae, despite a higher number of upregulated proteins in Aml as compared to Acc, the number of proteins with stronger expressional levels (log_1.5_ Ratio ≥ 3.5) in Aml was 11 and 16 in the Acc (Additional file 1: Table S1; Figure 5).

Proteins with stronger expression levels (log_1.5_ Ratio ≥ 3.5) in the Aml compared to Acc were 5 to 1, 10 to 6, and 4 to 0 on day 1, day 3 and day 5 pupae, respectively (Additional file 1: Table S1; Figure 5).

### Metabolic pathway analysis

**Figure 6:**
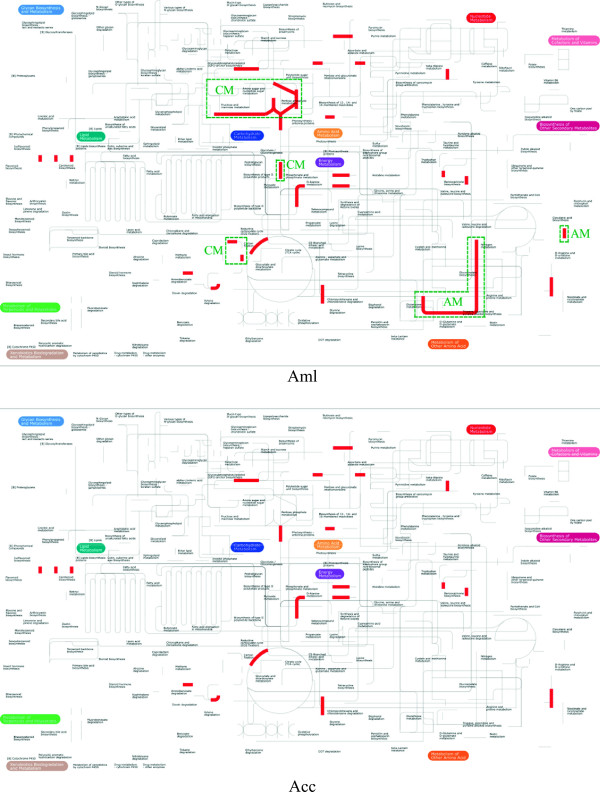
**Upregulated metabolic pathways as dictated by** ***A. m. ligustica*** **and** ***A. c. ceran*** **honey bee worker larval and pupal hemolymph across the different developmental stages.** Proteins with significantly increased abundance (*p* < 0.05, fold change >1.5) in worker bee larvae and pupae on day 1, 3 and 5 of *A. m. ligustica* (A) and *A. c. cerana* (B) were mapped to KEGG pathways (labeled in red with brighter shades) using iPath v.2.0. The different metabolic pathways between the two bee species, carbohydrate metabolism (CM) and amino acid metabolism (AM) are labeled with green dashed boxes.

To visualize the functional differences of hemolymph between the two bee species, differentially abundant proteins (Additional file 1: Table S1) were mapped to KEGG-derived metabolic pathways using iPath2.0 [24]. Only those honey bee proteins with assigned functions (i.e. KEGG KO or Enzyme EC number) could be mapped. The hemolymph proteins were matched to 57 metabolic map elements (37 in Aml versus 20 in Acc, redundant entries included) and allowed for a more pathway-centric view of the functional reactions specific to each honey bee species (Figure 6A and 6B). Proteins exhibiting increased abundance in the two bee species were represented as red edges on the KEGG. Even though similar coverage of the metabolic networks was observed for each bee species, such as citrate circle, alanine metabolism, nicotinate and nicotinamide metabolism, the most revealing feature of these maps was the existence of molecular networks that were characteristic of a specific bee species. Four unique pathways were mapped to hemolymph of Aml, which were mainly involved in metabolism of carbohydrates and amino acids (Figure 6A).

### Protein-protein interaction (PPI) analysis

**Figure 7:**
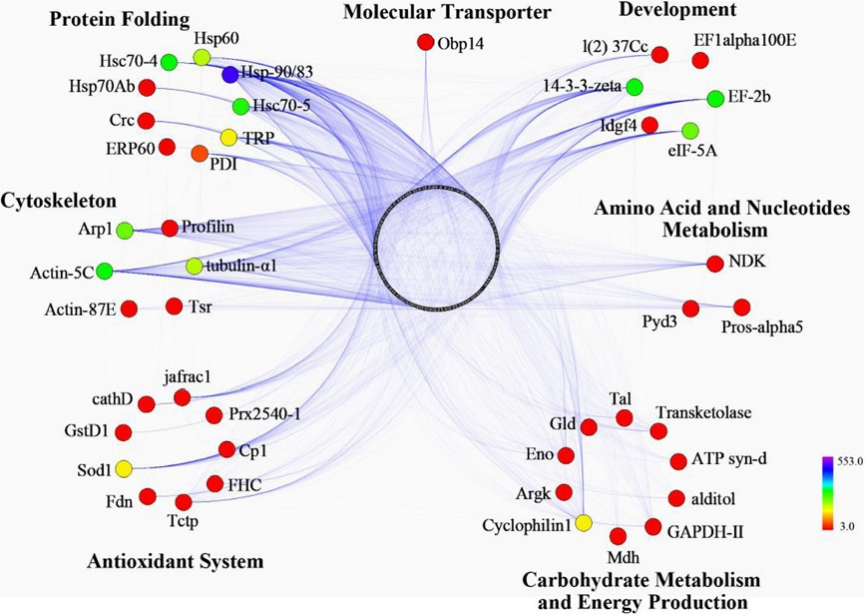
**Protein-protein interaction (PPI) network of the differentially expressed proteins from** ***A. m. ligustica*** **and** ***A. c. cerana*** **worker larval and pupal hemolymph at the different developmental stages.** PPI are predicted using I2D and visualized using Navigator software. Circles represent differentially expressed proteins connected in the network with more than 3 interaction degrees. Blue lines indicate interactions between proteins. The intensity of the interaction degree is indicated by color gradient as noted on the key bar on the lower right side of the figure.

Of the 74 differentially expressed proteins, 44 non-redundant proteins (36 in Aml and 18 in Acc), with 6 GO terms, were linked to the PPI network as key node proteins (Figure 7). Proteins involved in carbohydrate metabolism/energy production had the highest proportion in the network (22.7% or 10 proteins). Of these, 4 proteins (transketolase, GAPDH-II, ATP-syn-d, and MDH) were upregulated both in Aml and Acc, 5 proteins (Tal, argk, eno, gld, and alditol) were upregulated in Aml, and one protein (cyclophilin1) was upregulated only in Acc. Two groups of proteins, antioxidant system and protein folding, were the second most represented (20.5% or 9 proteins). In the antioxidant system group, 4 proteins were upregulated only in Aml (fdn, cp1, cathD, and jafrac1), and 4 proteins were upregulated in Acc (gstD1, Tctp, FHC, and Prx2540-1), and one protein was upregulated in both species (sod1). Of the proteins involved in folding activities, 7 were upregulated in Aml (Hsp60, Hsp70Ab, ERp60, Hsc70-4, Hsc70-5, PDI, and Crc) and 4 proteins were upregulated in Acc (Hsp60, TRP, Hsp90/83 and Hsc70-4). Of the 6 proteins (13.6%) networked in the cytoskeleton, 5 proteins were upregulated in Aml (Profilin, tubulin-α1, Arp1, actin-87E, and tsr), while 1 protein was upregulated in both (actin-5c), and no proteins were upregulated only in Acc. Similarly, of the 6 proteins (13.6%) involved in development, 5 were upregulated in Aml (14-3-3zeta, ef-2b, eif-5A, l(2)37Cc, and ef1-alpha100E), whereas one (Idgf4) was upregulated in both, and no proteins were upregulated only in Acc. Three proteins associated with the metabolism of amino acids and nucleotides were linked to the network, of which 2 were upregulated in both (NDK/Awd and Pyd3), and one protein was upregulated in Aml (pros-alpha5). The molecular transporter was networked with 1 protein that was upregulated in Aml (OBP14).

### Western blot

**Figure 8:**
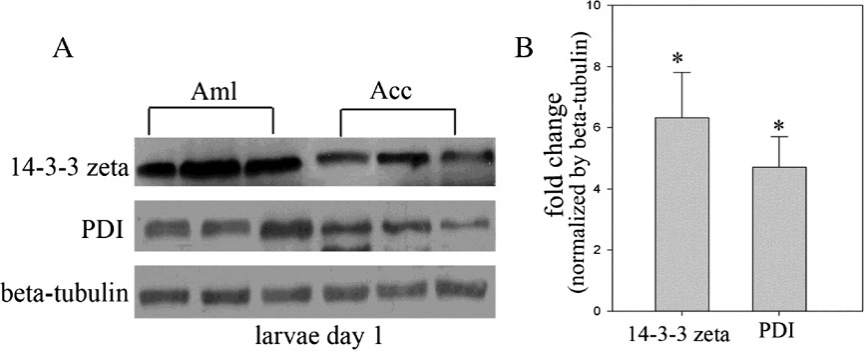
**Western blot analysis of 14-3-3 zeta and PDI.** Hemolymph proteins samples of honey bee worker (*A. m. ligustica* and *A. c. cerana*) larvae and pupae on days 1, 3 and 5 were subjected to SDS-PAGE followed by Western blot analysis. 14-3-3 zeta and PDI were detected using corresponding antibodies. Beta-tubulin was used as reference control. **(A)** The Western blot images of 14-3-3 zeta, PDI and beta-tubulin. **(B)** The relative fold change of 14-3-3 zeta and PDI (normalized by beta-tubulin). The gray bars represent relative fold change of protein expression. Error bar is standard deviation (*p* < 0.05).

The protein expressional tendency of 14-3-3 zeta and PDI was further verified by western blot analysis. The results were consistent with the results of 2-DE analysis (Figure 8).

## Discussion

To address biological discrepancies of the hemolymph underpins the ontogenesis of larvae and pupae between the western and eastern honey bees at a molecular-level, we used a time-resolved brood body weight and hemolymph proteomics comparison. The western bee has a significantly larger body weight than that of eastern bees (Figure 1), and remarkable differences in levels of protein expression (Figure 5), metabolic pathways appear when compared to its eastern counterpart (Figure 6). Hemolymph is of equal importance for the two bee spices in energy storage, odor communication, and antioxidation in terms of nutritional supply and immune defense, indicated by the elevated expression of different proteins. However, stronger expressions of proteins related to folding, cytoskeletal, and developmental activities as well as highly induced energy producing pathways in western bees may support their big body size in high nutritional demand and boost the immune defense [1, 9]. The high intensity of involvement of key node proteins in the PPI network (Figure 7) provides the potential target proteins for the future functional analysis of hemolymph.

### Energy stores are of equal importance for the two bee species in providing nutrition, boosting immune defense and brood growth

During the first 5–6 days after hatching, the larvae experience a striking 1500-fold gain in body weight. Within this period, the fast growing larvae feed plentifully, preparing themselves for the high energy demands of pupation, when no feeding will occur but the internal organs and body regions are shaped [25]. Although specific larval organs do not develop until the late larval stages, the fat body is the most represented of solid tissues, accounting for 65% of body mass, during the larval stage [26]. This fat body synthesizes large amount of hexamerins, which act as energy stores, and lipoproteins, which drive larval growth [2]. These proteins appear in the hemolymph near the end of the larval developmental stage [27]. Besides having a nutritional role, hexamerins are also used to build adult structures [28] in latter larval development [29]. Similar to hexamerins as energy stores, MRJPs can provide a high amount of essential amino acids to satisfy the nutritional demands of the growing larvae [3]. MRJPs may also function as immune mechanisms to ensure normal growth of bee brood. They defend against invading pathogens such as *Paenibacillus larvae* for the young honey bee larvae (the first 48 h) [3]. Recent evidence indicates that MRJP1 can modulate larval cast differentiation [30] and is involved in creation of filamentous structures in the honey bee brain [31]. The importance of these proteins for the larval growth is evidenced by the abundantly expressed hex70b (log_1.5_ Ratio ≥ 3.5 in day 5 larvae and day 1 pupae in Aml), hex110 (log_1.5_ Ratio ≥ 3.5 in day 5 larvae in both Aml and Acc), VHDL and MRJPs (MRJP1, 2, 3 and 5) (MRJP 1 and MRJP 2, log_1.5_ Ratio ≥ 4.5 in day 1 Aml larvae). These suggest that proteins related to energy storage play multifaceted roles as nutrients, defense agents and building blocks of the larval body to boost the normal development of larvae, which is in line with the previous reports [3, 32]. One of the advantages of 2-DE technology is that the modified proteins are visualized as a string of different protein spots with distinct isoelectric points (p*I*) on the gel images [33] as shown in our previous study on the post-translational modification (PTM) of royal jelly [34]. So the MRJPs, i.e. MRPJ1, MRJP2 and MRJP3, identified by different spots (Figures 2 and 4), may be the consequence of methylation or phosphorylation modification which can increase the nutritional efficiency of these proteins to the larvae [34]. Noticeably, the fact that we identified more hemolymph protein spots of MRJP1 in Acc than in Aml may reflect the different PTM status of this protein in the hemolymph of the two bees. It has been reported the glycosylated MRJP2 can effectively inhibit *P. larvae* infection [35]. Thus it is believed that different bee species may have different adaptive mechanisms of protein modification to meet the nutritional and immune demands for their own larvae. Similar to MRJPs, the identified high number of hexamerins in different species may serve the same purpose of increasing the content of amino acids as efficient nutrients or body building materials for honey bees [36].

### Protein-folding is important for the two bee species to promote brood development and immunity

Protein-folding chaperones and heat shock proteins (Hsps) are crucial in protein quality control machinery for achieving a proper functional shape or conformation during and after protein biosynthesis [37]. Hsps have been reported to facilitate nascent closure during the early larval development of the honey bee [38]. The high level expression of different proteins related to folding in both bees species may promote the fast growth of the brood [37] by facilitating nascent protein folding and maintaining cell shape and normal tissue development [38]. Noticeably, during the pupal stage, all of the identified proteins associated with protein folding, are more highly expressed in Aml than Acc. This stronger expression in Aml suggests that their larger body size may require large amounts of protein building blocks to sustain normal organogenesis [39, 40], which is in line with the heavier brood body weight in Aml than in Acc (Figure 1). Hsps also act as part of a defense mechanism, however, such as during larval and pupal infection with *P. larvae* or *Bacillus* [41, 42]. The enhanced expression of Hsps and calreticulin in the larval hemolymph of the two bee species may indicate their involvement in the immune system activation against the invasive pathogens [10, 43]. These include Hsc70-4 in day 3 larvae in Acc (log_1.5_ Ratio > 4), Hsp83/90 in day 3 and day 5 larvae in Acc (log_1.5_ Ratio > 3.5), protein disulfide-isomerase (PDI) in day 1 and day 3 larvae in Aml (log_1.5_ Ratio ≥ 4). Even though protein-folding proteins are expressed at different times and differently between species during the larval stages, they may achieve the same purposes in quality control of nascent protein synthesis and in immune defense.

### Energy producing pathways are enhanced in Aml to respond to its large body size

The growing larvae have high energy needs. Fat accumulation is of vital importance, demonstrated both by the high representation of fat body tissue relative to the whole organism at this stage, and by the buildup of lipophorins. Because larval food contains only 4% of lipids by weight, the accumulated fats in the larvae are mainly the consequence of *de novo* synthesis [44]. These endergonic biosynthetic processes must result in high demand for ATP. The energy producing proteins involved in metabolism of carbohydrates, fatty acids, and amino acids work together to meet these demands. This is reflected in our data that varieties of energy producing proteins were upregulated by both Aml and Acc (Figure 4). Of these upregulated proteins, they were mainly related to carbohydrate metabolism, such as aldh, transketolase, gapdh-2, enolase, arginine kinase and ATPsyn-d, they are principally involved in glycolytic activities, citrate circle, pentose phosphate metabolism and so forth (Additional file 1: Table S1). Proteins related to fatty acid metabolism are important for transferring energy from mitochondria to areas of high energy demand as the honey bee develops [45]. During development proteins such as FABP and Rfbag act as key regulators in the uptake and enzymatic activation of fatty acids to generate and transport energy [46]. Proteins implicated in metabolism of amino acids and nucleotides (pros-alpha5 and NDK/Awd) have roles not only for the metamorphosis [47] and tissue construction of honey bee [27], but also for delivering amino acids for the synthesis of new proteins and development processes of some organs/glands [48]. They are important for critically triggering the metabolic machinery of glycolysis/gluconeogenesis by the conversion of food into chemical energy.

Noticeably, the observed high number of energy producing proteins with stronger expression in Aml, such as GAPDH-II and Arp1 (log_1.5_ Ratio ≥ 4) in Aml of day 3 larvae, PMCA with log_1.5_ Ratio ≥ 3.5 in Aml of day 5 larvae, Ald and GAPDH-II (log_1.5_ Ratio ≥ 3.5) in Aml of day 1 pupae, Mdh (log_1.5_ Ratio ≥ 3.5) in Aml of day 5 pupae (Figure 5), and more strongly induced energy-producing pathways of metabolism of carbohydrates and amino acids (Figure 6), suggest that the large body size requires enhanced metabolic energy for fat body building so as to respond the high demand during organogenesis [3].

### Aml requires a high expression of cytoskeletal and developmental proteins to boost growth

Cytoskeletal proteins play central roles in both intracellular transport and cellular division [49]. For example, the actin protein is dynamically remodeled, and this reorganization is regulated by actin-binding proteins in response to intracellular and extracellular signals that stimulate cell division and differentiation [50]. Tsr is involved in the control of actin-based motility processes and in the enhanced removal of ADP-bound actin monomers from the pointed end of an actin filament [51] through restriction of the actin polymerization [52]. Almost all of the identified cytoskeletal proteins in the Aml larvae were upregulated, (Actin-5C, tubulin-α1, and tubulin β1 with log_1.5_ Ratio ≥4.5), and this suggests large body size has greater requirements in order to accomplish cell and tissue building relative to the Acc.

As in other organisms, the development of honey bee brood requires growth factors to modulate growth. During the larval-pupal metamorphosis of holometabolic insects, the larvae undergo remarkable physiological changes to prepare for upcoming pupation and metamorphosis, and most of the organs and tissues are reshaped in the pupae. We identified development related proteins in high abundance in the hemolymph of the two species (such as ef1alpha 100E, 14-3-3-zeta, l (2)37Cc, log_1.5_ Ratio ≥ 3.5 in day 5 larvae in Aml and eif-5A and idgf4, log_1.5_ Ratio ≥ 3.5 in day 3 and 5 larvae in Acc). Idgf4 has a role in stimulating proliferation and polarization of imaginal disc cells [21] and in enhancing larval to pupal development where specific limbs and organs grow from imaginal discs containing highly differentiated cells [32]. The 14-3-3 protein family contributes to a wide variety of important signal transduction pathways that control cell cycles, apoptosis, and programmed gene expression [53]. Lethal (2) 37Cc (l(2)37Cc) has roles for the development of larvae and the hypopharyngeal gland in the honey bee [38, 54, 55]. Eif-5A has key roles in cell proliferation and cell-cycle regulation [23], and also in protein synthesis [29]. In general, the elevated expression of proteins related to development in the brood of both bee species implies that the developing larvae and pupae require the upregulation of above biosynthetic pathways related proteins to stimulate their growth. The stronger expression of the proteins associated with development in Aml suggests the larger body size requires highly involved growth factors to boost the growing brood.

### Aml and Acc have different mechanisms of odor communication and antioxidation

Hemolymph plays key roles in immune defense and energy storage, but it also delivers various molecules throughout the body, making it is an ideal system for monitoring changes in other pathways. Larvae have the capability to respond to outside stimuli and inside regulatory cues such as odorant-based communication and immune defense [56, 57]. This is reflected in our data that both bee species observed abundant expression of different odorant binding proteins (OBPs) in the hemolymph (OBP13, log_1.5_ Ratio ≥ 3.5 in day 3 pupae in Aml and Acc; OBP14/19d, log_1.5_ Ratio ≥ 3.5 in day 5 larvae and day 3 pupae in Aml), and an iron transporting protein (transferrin 1, log_1.5_ Ratio ≥ 3.5 in day 5 larvae in Aml). In all, the abundantly expressed molecular transporters over the larval and pupal stages may suggest the western and eastern bees have developed different communication and immune mechanisms to adapt to their respective ecological ranges.

As a living organism, honey bees maintain complex systems of multiple types of antioxidants to protect them from oxidative damage [58]. The identified different antioxidant proteins with high abundance in both bees (jafrac1 and FHC, log_1.5_ Ratio ≥ 4 in day 3 larvae in Acc; Tctp, log_1.5_ Ratio ≥ 3.5 in day 5 larvae in Acc; fdn and cathD, log_1.5_ Ratio ≥ 2.5 in Aml) suggests that the western and eastern bees have shaped the different antioxidant strategies to protect their respective brood from oxidative damage during the development of brood.

## Conclusions

Our time-coursed proteome comparisons of hemolymph between the western and eastern honey bee across various stages of brood development signify that the two bee species have different strategies to fit their different biological properties, shown by changes of the hemolymph proteome and energy producing pathways. Generally, hemolymph is important for both bee broods as nutrients and immune defense by the upregulated proteins associated with energy storage, protein folding, odor communication, and antioxidation. However the enhanced expressions of proteins implicated in folding, cytosckelal functions, and development as well as additionally activated energy producing pathways in western bees is in response to their big body size for the high nutritional demand and boosting the immune defense. The results also provide a valuable resource as a starting point for future functional research on the specific hemolymph proteins or pathways related to the differential phenotypes or physiology.

## Methods

### Chemical reagents

All reagents were analytical grade or better. All of the chemicals used for 2-DE were purchased from Sigma (St. Louis, MO, USA) except Biolyte and immobilized pH gradient (IPG) strips which were from Bio-Rad (Hercules, CA, USA), and modified sequencing grade trypsin, which was purchased from Roche (Mannheim, Germany). Other chemicals not mentioned here are sourced in the text.

### Biological samples

We sampled from five colonies of each species *Apis mellifera ligustica* (Aml) and *Apis cerana cerana* (Acc), maintained at the Institute of Apicultural Research, Chinese Academy of Agricultural Science, Beijing, China. Each queen was confined in a single wax comb frame containing worker cells for 6 h in each colony in order to collect the larvae of known ages. After releasing the queen from the cage, the eggs contained in the frame were maintained in the honey bee colony for further development. The hemolymph samples were collected from the larvae on days 1, 3, and 5 and from the pupae on days 1, 3, and 5, following our previously published protocol [9]. All the larvae and pupae were washed twice with phosphate buffered saline (PBS) before the hemolymph was collected in order to remove royal jelly contamination fed by nurse bees in the comb cells. The body weight of the collected larvae and pupae was measured first. Then, hemolymph was collected from the larvae aged days 1–3 by carefully piercing their skins (avoiding deep cuts so as to not to cause organ damage). For hemolymph collection from the larvae aged days 4–5 and all the pupae aged days 1–5, a disposable glass micro-capillary pipette (5 μL) was inserted to one side (two-thirds down from head in the body) of the larva and pupa, avoiding deep cuts and drawing hemolymph liquid by capillary action. Over 200 larvae and pupae were sampled in each replication from both bee species and an average of 250 μL of hemolymph was collected per sample. All the collected samples were stored at -80°C until used. For simplicity and following the tendency of protein changes, larvae and pupae aged days 1, 3, and 5, respectively, were sampled for the analysis.

### Protein extraction and Two-dimensional Gel electrophoresis (2-DE)

Protein extraction was done in accordance with Deregie et al. [9]. Protein concentration was determined following the Bradford method with a DU800 spectrophotometer (Backman Coulter, LA, CA). Bovine serum albumin (BSA) was used as standard, and absorption was measured at 595 nm.

Each 600 μg of larval and pupal protein sample from each of the six time-points from both Aml and Acc bees (with 3 biological replicates) was suspended in 90 μL of lysis buffer (LB, 8 M urea, 2 M thiourea, 4% CHAPS, 20 mM Tris-base, 30 mM dithiothreitol (DTT), and 2% Biolyte pH 3–10) and mixed with 360 μL of rehydration buffer (8 M urea, 2% CHAPS, 0.001% bromophenol blue, 45 mM DTT, 0.2% Biolyte pH 3–10). The first dimension isoelectric focusing (IEF) and second dimension electrophoresis as well gel equilibrations were performed according to Deregie et al. [9]. The gels were fixed for 3–5 h in 50% (v/v) ethanol with 10% (v/v) acetic acid, stained with CBB G-250 over night, and protein spots were visualized for the subsequent analysis.

### Image analysis

Three independent and reproducible 2-DE gel images from the samples at each time point were digitized at the resolution of 16 bit and 300 dpi using an Image Scanner III (GE Healthcare, Piscataway, NJ, USA). Gel images were subjected to analysis by Progenesis Samespot software (version 4, nonlinear Dynamics, UK) for image quality control, spot alignment, filtration, normalization and quantitation of spot volume. One image was selected automatically as the reference gel to do gel analysis. All gels were matched in an automatic mode and further manual editing was done to correct the mismatched and unmatched spots. The expression level of each protein spot was calculated in terms of its volume. Statistical analyses followed our previous method [9]. Only spots with at least 1.5-fold changes and *p* < 0.05 of volume were considered as statistically significant differences.

### Trypsin digestion and protein identification by Mass Spectrometry (MS)

Only the differentially expressed hemolymph protein spots were manually excised, the subsequent protein digestion and peptide extraction were undertaken according to Zhang et al. [34]. The digested protein spots were analyzed by liquid chromatography (LC) MS system (LC-Chip/ESI-QTOF-MS, QTOF G6520, Agilent Technologies), equipped with a capillary pump G1382A, a nano pump G2225A, an auto sampler G1377D, and the Chip Cube G4240A according to our previously described protocol [34]. MassHunter software (version B. 03, Agilent Technologies) was used to retrieve tandem mass spectra. Peak-list was generated by Mascot Distiller software (version 3.2.1.0, Matrix Science) prior to MS/MS data search. The data were stored in a combined mgf file and searched against sequence database generated from protein sequences of *Apis mellifera* (downloaded Nov, 2012, version 4.5 of the honey bee genome) augmented with sequences from other honey bee species, as well as from *Drosophila melanogaster* (downloaded Nov, 2012) and *Sacharomyces cerevisiae* (downloaded Nov, 2012), and a common repository of adventitious proteins (cRAP, from The Global Proteome Machine Organization, downloaded Nov, 2012), totaling 72672 entries, using in-house Mascot (version 2.3, Matrix Science, UK). The search parameters included carbamidomethyl (C) and oxidation (M) (selected as fixed and variable modifications, respectively); taxonomy: all; enzyme: trypsin; missed cleavages: 2; peptide tolerance: ± 50 ppm, MS/MS tolerance: ± 0.05 Da.

When an identified protein was matched to multiple members of a protein family or if a protein appeared under the same names and accession number, the match was made in terms of differential patterns of protein spots on 2-DE gels. Protein identification was accepted if it contained at least two unique peptides, had a Mascot score above the cutoff of 31, with 95% confidence.

### Bioinformatics

The identified proteins were annotated by searching against the Uniprot database (http://www.uniprot.org/) and Flybase (http://flybase.org/). Proteins were manually grouped on the basis of their GO terms of biological process. For a network-wide perspective of hemolymph metabolism, we employed the iPath2.0 [24] (http://pathways.embl.de) to navigate and explore the predicted KEGG metabolic pathways. Proteins exhibiting significant (*p* < 0.05 and fold change > 1.5) abundance differences were used for KEGG pathway mapping to visualize differences between both bee species on a more global, metabolic pathway-centric level.

To identify protein linkages in the protein-protein interaction (PPI) network, proteins identified from the larval and pupal hemolymph of the two bee species were analyzed by the Interologous Interaction Database (I2D) (http://ophid.utoronto.ca/i2d) [59], which integrated known PPI data sets of *D. melanogaster. Drosophila* datasets were used because it is the closest highly studied phylogenetic neighbor of the bee and has well annotated PPI database. PPI networks were annotated, visualized, and analyzed using NAViGaTOR (http://ophid.utoronto.ca/navigator/). Only proteins with more than three interaction degrees were considered as key node proteins, indicating a high degree of interaction in the network.

### Western blot analysis

To verify the results of differentially expressed proteins at the protein level, 14-3-3 zeta and PDI were selected for western blot analysis. The primary antibodies were rabbit polyclonal anti-14-3-3 zeta and PDI (Abcam, Cambridge, MA, USA) at dilutions of 1:3000 and 1:2000, respectively. The secondary antibody was horseradish peroxidase-conjugated goat anti-rabbit at a dilution of 1:10000. Each 10 μg sample was separated by stacking (4%) and separating (12%) SDS-PAGE gels. Beta-tubulin was used as a reference and the western blot and data analyses were performed according to previous methods [12].

### Animal ethical use issues

Honey bees are not a regulated invertebrate. Therefore, no ethical use approval is necessary.

### Availability of supporting data

The data sets supporting the results of this article (Additional file 1: Table S1 and Additional file 2: Table S2) are included within the article and its additional files.

## Electronic supplementary material

Additional file 1: Table S1: Differerentially expressed hemolymph proteins during larval and pupal development stages of honeybee worker (*A. m. ligustica* and *A.c. cerana*) on days 1, 3 and 5, respectively. (XLSX 44 KB)

Additional file 2: Table S2: Protein abundance of the differerentially expressed hemolymph proteins during larval and pupal development stages of honey bee worker (*A. m. ligustica* and *A.c. cerana*) on days 1, 3 and 5, respectively. (XLSX 60 KB)
